# Coupled regulation effects of sanqi-pine intercropping systems on soil fertility and heavy metals mediated by ammonia-oxidizing microorganisms

**DOI:** 10.1186/s12870-026-09050-3

**Published:** 2026-05-20

**Authors:** Jingying Hei, Qianqian Dong, Yingjun Li, Rui Rui, Xiahong He, Shu Wang

**Affiliations:** 1 Yunnan Key Laboratory of Landscape Plant Resource Cultivation and Application, Southwest Forestry University, Kunming, 650224 China; 2 Yunnan Provincial Key Laboratory for Conservation and Utilization of In-forest Resources, Southwest Forestry University, Kunming, 650224 China

**Keywords:** Sanqi intercropping system, *P. armandii*, and *P. yunnanensis*, Ammonia-oxidizing microorganisms, Soil fertility, Heavy metals, Structural equation modeling

## Abstract

**Background:**

While the Sanqi-pine intercropping system enhances soil fertility and mitigates heavy metal accumulation, the dynamic interactions among soil properties, heavy metals, and microorganisms across different intercropping patterns and full growth stages remain poorly understood. Here, we established monoculture systems of *Pinus armandii* (Pa) and *Pinus yunnanensis* (Py), alongside their respective intercropping systems with Sanqi (PaS and PyS). Over a 24-month monitoring period, we determined key edaphic properties, heavy metal concentrations, and the abundance of ammonia-oxidizing microorganisms, aiming to systematically uncover the regulatory mechanisms underlying these interactions.

**Results:**

Both PaS and PyS systems significantly improved comprehensive soil fertility and effectively reduced the contents and pollution risks of heavy metals. The relative closeness of soil fertility increased by 19.35% and 18.18%, while the relative closeness of heavy metals decreased by 41.03% and 61.36% in the PaS and PyS systems, respectively. Notably, the pollution levels were all classified as GradeⅠ(clean/safe) in all the systems. Sensitivity analysis revealed that ammonium nitrogen (NH_4_^+^–N) and Cu were the core factors affecting soil fertility and heavy metal indices, respectively. Further correlation analysis demonstrated that soil fertility was significantly negatively related to heavy metal contents. Structural equation modeling (SEM) confirmed that ammonia-oxidizing bacteria (AOB) and the archaea–to–bacteria ratio (AOA/AOB) directly influenced Cu content by regulating NH_4_^+^–N transformation, thereby reducing heavy metal accumulation in the soil.

**Conclusion:**

This study confirms that both PaS and PyS systems improve soil fertility and mitigate heavy metal risks, while the PyS system exhibits superior comprehensive performance under its environmental conditions. These findings provide an important theoretical basis for the ecologically safe cultivation of Sanqi and the sustainable management of soil health.

**Supplementary Information:**

The online version contains supplementary material available at 10.1186/s12870-026-09050-3.

## Background

*Panax notoginseng* (Sanqi, its Chinese common name), a perennial herb of the family Araliaceae, is a rare and authentic medicinal plant endemic to China, widely used in the prevention and treatment of cardiovascular diseases and immunomodulation [1]. This species exhibits an obvious biological characteristic of preferring shade and humidity, and is suitable for establishing understory intercropping systems with various tree species, including *Platycladus orientalis*, *Schima superba*, *Alnus cremastogyne*, *Cunninghamia lanceolata*, *Pistacia weinmannifolia*, and *Eucalyptus robusta* [2]. Prior studies have demonstrated that, compared with broad-leaved tree species, intercropping systems of Sanqi with coniferous tree species such as *Pinus armandii*, *P. yunnanensis*, and *P. kesiya* can improve Sanqi quality, enrich soil beneficial microorganisms, and promote soil metabolites, and alleviate continuous cropping obstacles [3–6]. These positive regulatory effects are mainly attributed to the fact that coniferous trees optimize key indicators of soil fertility through multiple pathways, including litter decomposition, root exudate release, and rhizosphere microbial driving, thereby creating a stable and coordinated rhizosphere nutritional environment for Sanqi growth [2, 7, 8]. However, soil quality represents a dialectical unity of fertility level and environmental safety, and heavy metal accumulation risk is directly related to the quality and safety of traditional Chinese medicinal materials and the sustainability of planting systems [9]. Sanqi cultivation significantly improves soil fertility in Sanqi soil and reduces bioavailable heavy metals via immobilization in both Sanqi rhizosphere and *P. armandii* soils, without enhancing heavy metal accumulation in Sanqi medicinal parts [10]. At present, relevant studies mostly focus on single Sanqi-pine intercropping systems (SPI), lacking comprehensive evaluations of different SPI systems. Therefore, systematically analyzing the differences in soil fertility characteristics and heavy metal risks across various SPI systems is of great theoretical and practical guidance for scientifically selecting superior associated coniferous tree species and constructing a high-quality, and ecologically safe SPI system.

Geographical location [11], cropping patterns [10], cultivation duration [12], and seasonal variations [13] constitute the four pivotal environmental and management determinants that collectively govern the intricate dynamics of soil heavy metal accumulation and soil fertility evolution. Geographical location fundamentally determines the pedogeochemical background and migration potential of heavy metals; specifically, parent material acts as the core determinant [14], while topography facilitates metal enrichment in low–lying areas through hydrothermal redistribution [15]. Moreover, cropping patterns directly modulate heavy metal bioavailability and soil fertility by altering intra-system material cycles and energy flows. For instance, Sanqi–pine intercropping system effectively boosts soil fertility and reduces heavy metal levels [10], a positive effect primarily attributed to organic management practices. Furthermore, cultivation duration reflects the cumulative impact of anthropogenic activities; notably, prolonged cultivation leads to soil acidification and a significant rise in the total and bioavailable fractions of cadmium (Cd), copper (Cu), and zinc (Zn), posing potential risks to soil quality and crop safety [12]. Additionally, seasonal variations regulate heavy metal speciation and nutrients mineralization-immobilization process by driving periodic environmental fluctuations. Specifically, temperature and humidity dynamics impact soil redox potential (Eh), organic matter decomposition, and microbial activity, thereby inducing synchronous shifts in soil fertility and heavy metal availability [16, 17]. As mentioned above, soil environmental health emerges from the complex interplay of these synergistic or antagonistic processes. Therefore, it is of great significance and necessity to conduct soil environmental assessments from a multi-scale perspective—encompassing the entire plant growth period, long-term time series, and multiple regions—to fully comprehend the laws governing soil heavy metal accumulation and fertility evolution, and to ensure soil environmental safety.

Ammonia-oxidizing archaea (AOA) and bacteria (AOB), as the key drivers of nitrification in the soil nitrogen cycle, govern the transformation and availability of soil nitrogen by oxidizing ammonium (NH_4_^+^) to nitrite (NO_2_^–^) and nitrate (NO_3_^–^) [18]. Concurrently, AOA and AOB indirectly modulate the bioavailability, solubility, mobility, and toxicity of heavy metals by altering environmental physicochemical properties, particularly pH and redox potential [19]. Conversely, heavy metals exert toxic stress on the activity and community structure of AOA and AOB. Numerous studies have confirmed that Cd and copper Cu significantly inhibit soil nitrification processes and ammonia-oxidizing microorganisms [19, 20]. Notably, AOA and AOB differ markedly in their sensitivity to heavy metal pollution. AOB generally exhibit higher susceptibility to metals such as Cu, Zn, and Cd [21, 22]. In heavy metal-contaminated soils, the abundance and community composition of AOB are typically more severely inhibited, whereas AOA display stronger tolerance [23, 24]. Furthermore, a close interaction exists between soil heavy metals and soil fertility [10]. On the one hand, heavy metals can continuously exacerbate soil fertility degradation by inhibiting plant nutrient uptake [25], reducing soil enzyme activity [25], disrupting microbial community structure [26], depleting soil organic matter content [27], and deteriorating soil physicochemical properties [28, 29]. On the other hand, robust soil fertility can effectively mitigate heavy metal toxicity by hindering metal migration and diffusion [30], passivating heavy metal bioavailability [27], adsorbing heavy metal cations [31], and reducing metal solubility [32, 33]. In summary, AOA and AOB serve not only as the critical nexus connecting the soil nitrogen cycle with the geochemical behavior of heavy metals [34] but also as the core entry point for understanding nitrogen cycling disorders in contaminated soils and formulating remediation strategies [35].

Accordingly, this study selected two typical conifer intercropping systems composed of *P. notoginseng* (Sanqi) with *P. armandii* (PaS) and *P. yunnanensis* (PyS) as research subjects, with monoculture stands of *P. armandii* and *P. yunnanensis* as the control. Following a 24-month in-situ monitoring campaign, soil physicochemical properties, heavy metal concentrations, and abundance dynamics of ammonia-oxidizing microorganisms were systematically analyzed. Multiple statistical approaches, including the entropy weight-TOPSIS method, sensitivity index, Nemerow pollution index, and structural equation modeling (SEM), were employed to identify the understory intercropping patterns of Sanqi with optimal comprehensive benefits. The objectives of this study were to: (1) elucidate the divergent effects of intercropping patterns between different pine species and Sanqi on overall soil fertility and heavy metal contamination risk; (2) uncover the key regulatory roles of ammonia-oxidizing microorganisms in soil nitrogen transformation and heavy metal accumulation. These findings will lay a scientific foundation and offer technical guidance for the sustainable management of soil health and the ecologically safe cultivation of Sanqi.

## Materials and methods

### Study area and Sanqi cultivation

Four agroforestry systems, including *P. armandii* (Pa) vs. *P. armandii*-Sanqi (PaS) and *P. yunnanensis* (Py) vs. *P. yunnanensis*-Sanqi (PyS), were located in Xundian Hui and Yi Autonomous County, Kunming City (103.12°E, 25.28°N) and Bashang Town, Linxiang District, Lincang City (100.12°E, 23.40°N), Yunnan Province, China, respectively. The environmental characteristics of the two experimental sites were described below: The Pa and PaS site, located at an altitude of 2014 m, had an annual average temperature of 14.5℃ and relative humidity of 70%, and was dominated by *P. armandii* with an average tree height of 9.5 m and diameter at breast height (DBH) of 18 cm. In comparison, the Py and PyS site, situated at 2,181 m, featured an annual average temperature of 18.8℃ and a relative humidity of 80% with a low-latitude plateau mountain monsoon climate and red soil. This site was mainly covered by *P. yunnanensis* with an average tree height of 9 m and DBH of 17 cm.

In December 2021, experimental plots with slopes of 5–15° were established for the PaS and PyS agroforestry systems. Following the removal of stones and residual vegetation, the soil was tilled to a depth of 20–30 cm and amended with hydrated lime for pH regulation. Contour ridges were then constructed with a uniform height of 40 cm, tapering from 120 to 150 cm at the base to 80–100 cm at the top. One-year-old Sanqi seedlings were transplanted into these ridges at a depth of 3–5 cm with a spacing of (10–15) cm × (10–15) cm, and subsequently backfilled with 2–5 cm of soil. To conserve soil moisture, a 3–5 cm thick layer of pine needle mulch was applied to the soil surface. Standardized intercropping and management procedures were implemented as outlined in Hei et al. [1, 5]. Only commercial and widely used Sanqi varieties were planted in the artificial pine cultivation bases, so no permits were required.

### Soil sampling

A randomized block design was employed across the four agroforestry systems, establishing 12 plots (4 treatments × 3 replicates), each measuring 10 m × 10 m. Soil sampling was conducted monthly from January 2022 to December 2023, spanning a period of two years. In total, 288 soil samples were collected (4 treatments × 3 replicates × 24 months). For each plot, soil was collected from five points using the five-point sampling method and then thoroughly mixed to form one composite sample per replicate [5]. In the PaS and PyS plots, ten healthy Sanqi plants were selected. The intact root systems were excavated, and the soil loosely adhering to the root surfaces was gently shaken off and collected as rhizosphere soil. For the collection of pine rhizosphere soil, the soil was carefully excavated with a small trowel to avoid damaging the roots. Fine roots (2–5 mm in diameter) were identified, and the soil tightly attached to the root surface (approximately 0.5–1 mm thick) was gently detached using sterile tweezers. Concurrently, bulk soil (non-rhizosphere soil) samples were collected from a distance of 1.5–2.5 m from the pine trunks (an area devoid of fine pine roots) using a sterile soil auger at a depth of 0–20 cm. Immediately frozen in liquid nitrogen and transported to the laboratory, all samples were sieved with the same particle size (0.149 mm) and dried for the determination of edaphic factors and heavy metal content, or stored at − 80 °C for the analysis of ammonia-oxidizing microorganisms.

### Determination of edaphic factors and heavy metal indicators

The soil water content (SWC) was quantified using the gravimetric method, which involved drying soil samples at 100 °C for 24 h until a constant mass was achieved. To determine soil pH, a soil-to-water suspension (1:5 w/v) was prepared and measured with a pH meter. Total nitrogen (TN) and total phosphorus (TP) were determined in H_2_SO_4_–HClO_4_-digested solutions, whereas ammonium nitrogen (NH_4_^+^−N) and nitrate nitrogen (NO_3_^−^−N) were determined in KCl extracts, using a continuous-flow analyzer (Seal Auto Analyzer AA3, SEAL Analytical, Germany). The contents of total potassium (TK), Zn, manganese (Mn), Cu, lead (Pb), chromium (Cr), and Cd were determined via flame atomic absorption spectrophotometry (Labomed Inc, USA). Soil organic matter (SOM) was assessed based on the consumption of potassium dichromate (K_2_Cr_2_O_7_) during oxidation, and soil organic carbon (SOC) was subsequently calculated by dividing the SOM value by the conversion factor of 1.724.

### Quantitative Real-Time PCR (qPCR) analysis

Quantification of AOA and AOB gene abundances was conducted via quantitative PCR (qPCR) on a LightCycler 480 II Real-Time PCR System (Applied Biosystems, USA). Gene-specific primers are detailed in Table S1. The amplification mixture (20 µL) comprised 10 µL of 2×SYBR Green Premix Pro Taq HS qPCR Kit, 0.5 µL of each primer (10 µmol·L^− 1^), 1 µL of template DNA (50 ng·µL^− 1^), and 8.0 µL of ddH_2_O. The thermal cycling protocol included an initial denaturation at 95 °C for 3 min, followed by 40 cycles of 95 °C for 10 s, annealing at 57/55°C for 30 s, and extension at 72 °C for 30 s.

Standard curves were constructed using ten-fold serial dilutions (10^− 1^ to 10^− 7^) of plasmid DNA harboring the target gene inserts. These curves yielded R^2^ values exceeding 0.99, with regression equations of Y = − 3.105x + 42.918 and Y = − 3.291x + 45.046. All reactions were performed in triplicate alongside non-template controls to monitor potential contamination. Amplification efficiencies fell within the 90–110% range, and melting curve analysis verified the specificity of the amplicons.

### TOPSIS, Nemerow pollution index and sensitivity analysis

The Technique for Order of Preference by Similarity to Ideal Solution (TOPSIS) model was employed to conduct a comprehensive evaluation of soil fertility and heavy metal content, following the calculation procedures detailed by Liu et al. [10]. Initially, all indicators were classified as beneficial indicators (higher values represent better soil quality) and cost indicators (lower values represent better soil quality). Original data were dimensionless standardized to eliminate dimensional differences and mitigate the interference of asymmetric data distribution. Subsequently, a standardized matrix was constructed, and the positive ideal solution (maximum value of each indicator) and negative ideal solution (minimum value of each indicator) were determined based on all sample data. The Euclidean distance of each sample to the positive and negative ideal solutions was then calculated, respectively. Finally, the relative closeness coefficient was obtained to quantify the comprehensive level of soil fertility and heavy metal content. The Nemerow pollution index was employed to comprehensively assess the degree of heavy metal pollution in soil samples, where both the single-factor pollution index (Pi) and the Nemerow composite pollution index (NPi) were calculated in accordance with the formulas proposed by Liu et al. [10]. Sensitivity analysis was conducted to identify the relative contribution of parameters and verify the rationality of indicator selection; a higher sensitivity index implies a more significant impact of the corresponding parameter on the assessment results, and its specific calculation method was also adopted from Liu et al. [10].

### Statistical analysis

The Shapiro-Wilk test and Levene test (*P* < 0.05) were used to verify the normality of distribution and homogeneity of variance, respectively. SPSS 26.0 software (SPSS, Inc., Chicago, IL, USA) was employed to perform two-way analysis of variance (ANOVA) on indicators including edaphic factors, heavy metal concentrations, relative closeness, single-factor pollution index, and Nemerow pollution index. Multi‑way ANOVA was used to evaluate the effects of geographical location, year, seasonal dynamics, and planting pattern on soil fertility and heavy metal characteristics. Only main effects and two‑way interactions were interpreted, while high‑order interactions were not emphasized due to limited statistical power. Effect sizes are presented as partial eta squared (η_p_²), which indicates the variance explained by each factor independent of other model terms. Significance level was set at *P* < 0.05. Mantel test analysis was established using the “ggplot2”, “linkET”, and “psychstats” packages in the R language, while linear regression models were constructed with the “geosphere” and “vegan” packages. The goodness-of-fit index (GFI) was used to evaluate the SEM.

## Results

### Variations in edaphic factors and soil fertility

**Fig. 1:**
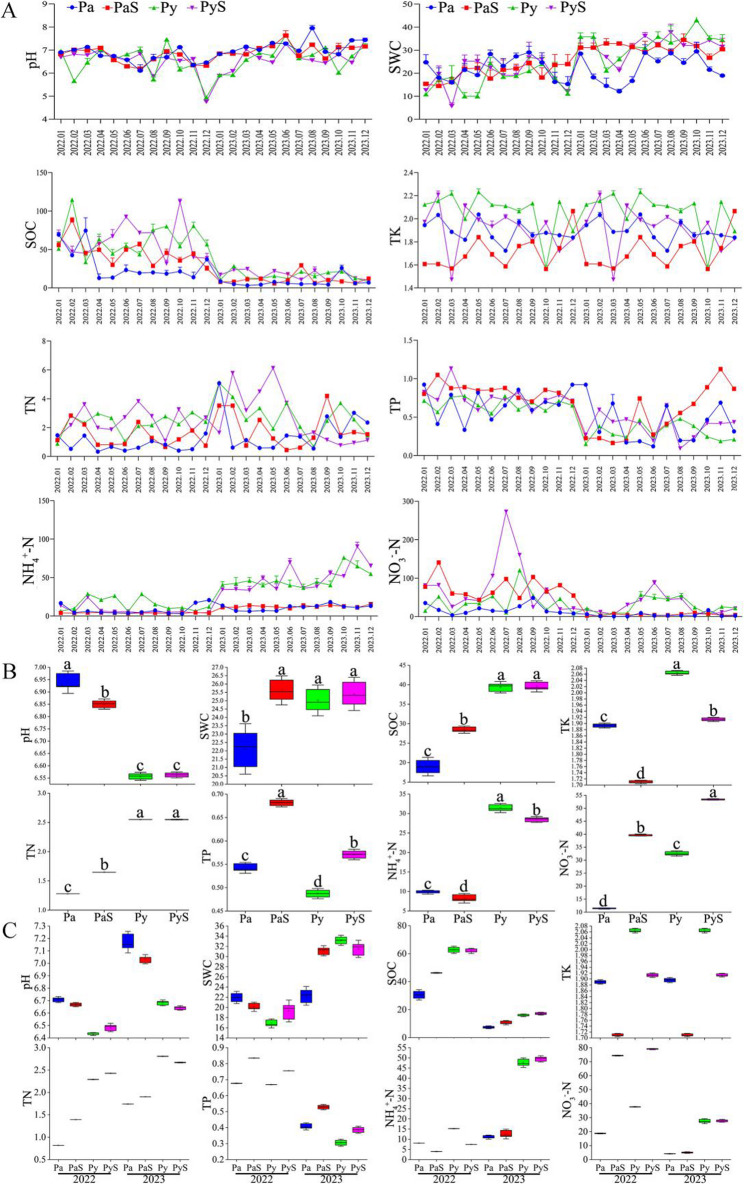
Monthly variations in edaphic factors (pH, SWC, TN, TP, TK, SOC, NH_4_^+^−N, and NO_3_^−^−N) concentrations from January 2022 to December 2023 (**A**), and their average distributions across treatments (**B**) and annual averages in 2022 and 2023 (**C**) under different cropping systems. Different lowercase letters indicate significant differences among treatments (*P* < 0.05)

Compared with Pa soil, PaS soil showed significant increases in NO_3_^−^−N, SOC, TN, TP, and SWC by 247.33%, 49.84%, 28.91%, 25.93%, and 15.88%, respectively, whereas NH_4_^+^–N, TK, and pH decreased by 13.96%, 9.53%, and 1.30%, respectively. Compared with Py soil, PyS soil displayed elevated NO_3_^−^−N (63.83%) and TP (16.33%) contents, accompanied by reduced NH_4_^+^–N (9.35%) and TK (7.28%) contents, with no significant differences detected in SOC, TN, pH, or SWC (Fig. 1). Furthermore, pH, SWC, TN, and NH_4_^+^–N were all higher in the 2nd year than in the 1st year, while SOC, TP, and NO_3_^−^−N exhibited the opposite pattern; TK content showed no significant difference between the two years.

**Fig. 2:**
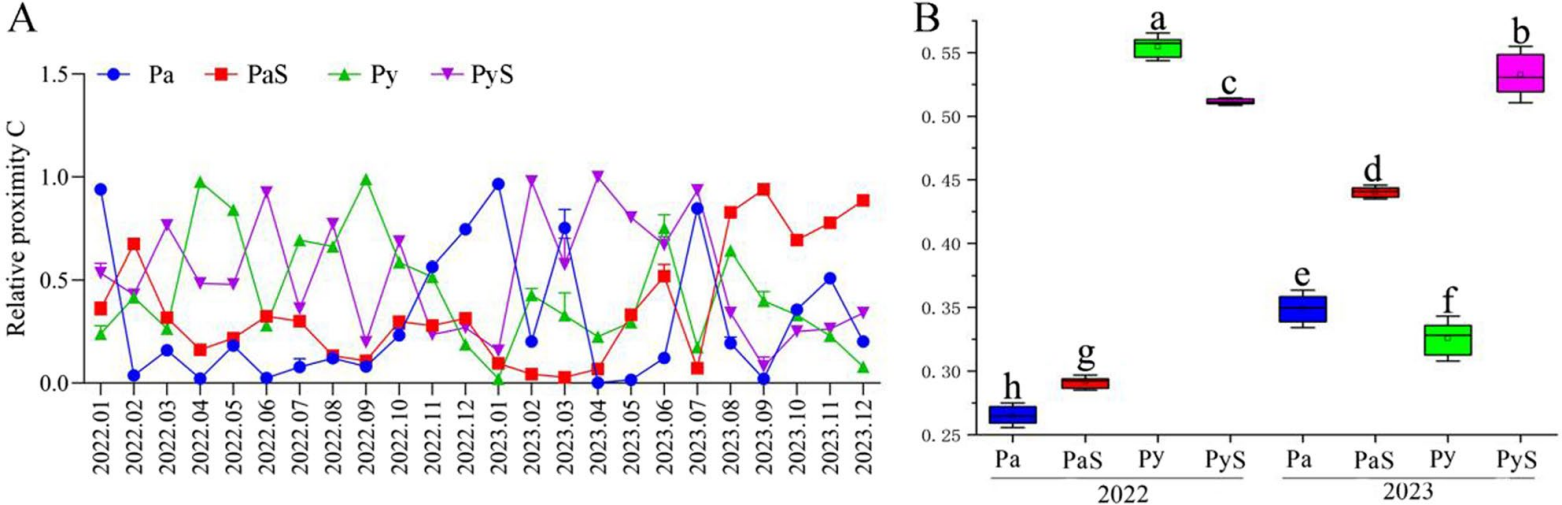
Monthly variations (**A**) and annual averages (**B**) in soil fertility relative closeness from January 2022 to December 2023 under different cropping systems. Different lowercase letters indicate significant differences among treatments (*P* < 0.05)

Compared with Pa soil, the average relative closeness of soil fertility in PaS soil was significantly increased by 19.35%, with respective increases of 7.41% and 25.71% in the 1st and 2nd years. Similarly, the relative closeness of soil fertility in PyS soil was significantly elevated by 18.18% compared to Py soil, characterized by a 7.27% decrease in the 1st year and a substantial 60.61% increase in the 2nd year (Fig. 2; Table S2). Results from main and interaction effects indicated that geographical location (η² = 0.107, *P* = 0.000) was the primary factor influencing the relative closeness of soil fertility, followed by planting pattern (η² = 0.027, *P* < 0.01) (Table S3). Neither year (η² = 0.000, *P* = 0.809) nor season (η² = 0.000, *P* = 0.991) exerted a significant main effect. In contrast, the interaction between geographical location and season had the most pronounced impact on soil fertility (η² = 0.235, *P* = 0.000) (Table S3).

### Changes in soil heavy metals and main/interaction effects analysis

**Fig. 3:**
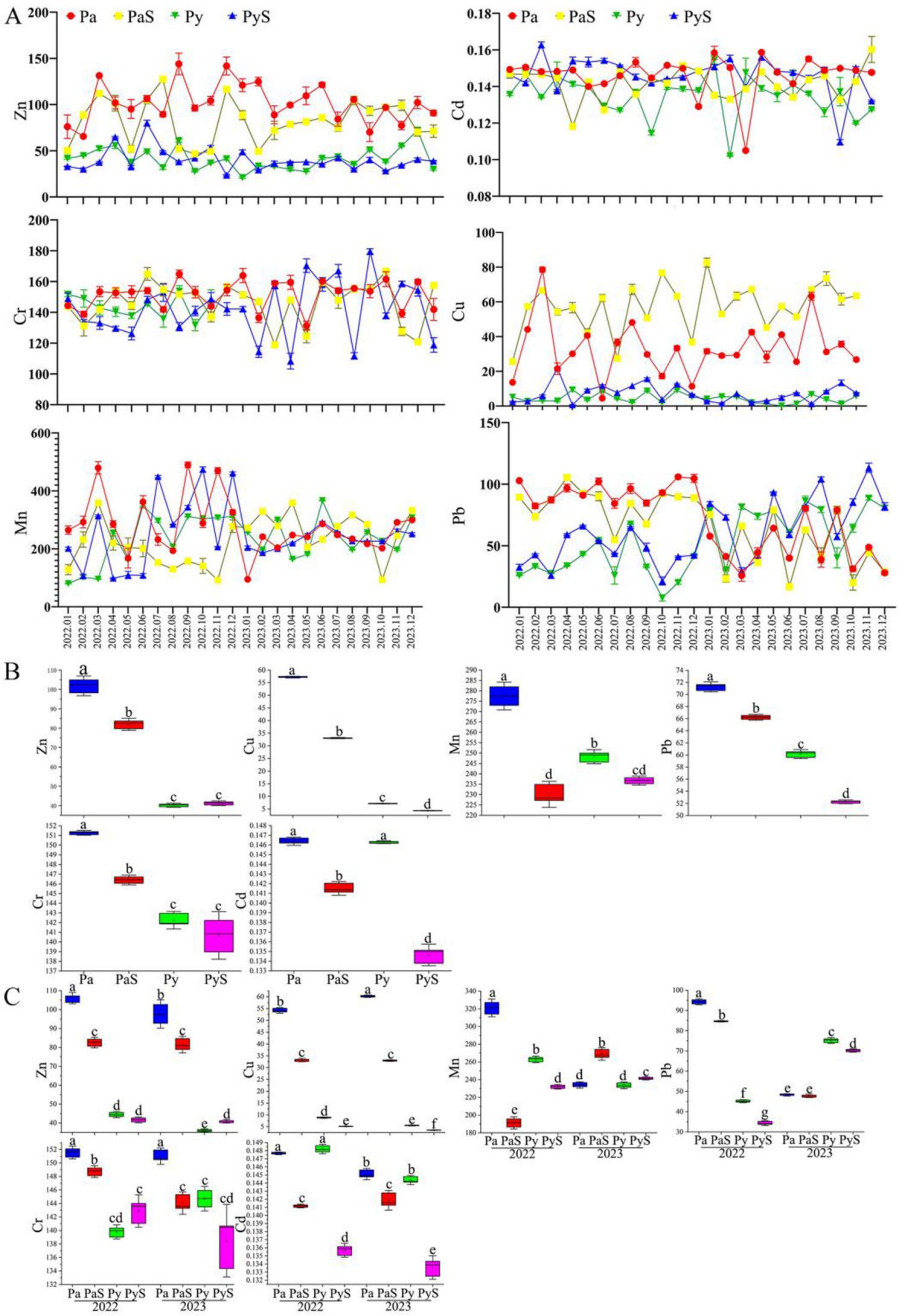
Monthly variations in soil heavy metal (Zn, Cr, Mn, Cd, Cu, and Pb) concentrations from January 2022 to December 2023 (**A**), and their average distributions across treatments (**B**) and annual averages in 2022 and 2023 (**C**) under different cropping systems. Different lowercase letters indicate significant differences among treatments (*P* < 0.05)

**Fig. 4:**
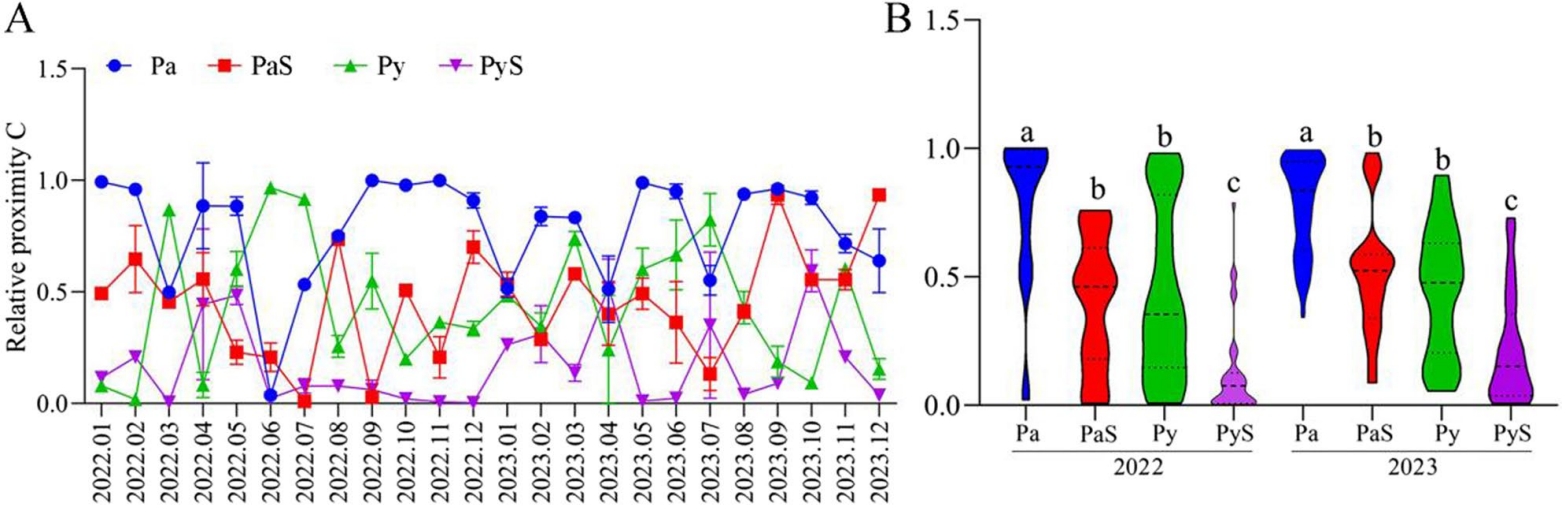
Monthly variations (**A**) and annual averages (**B**) in soil heavy metal relative closeness from January 2022 to December 2023 under different cropping systems. Different lowercase letters indicate significant differences among treatments (*p* < 0.05)

Compared with Pa soil, PaS soil exhibited reductions in the contents of Cu, Zn, Mn, Pb, Cd, and Cr by 42.19%, 19.49%, 17.08%, 7.07%, 3.33%, and 3.22%, respectively. Relative to Py soil, PyS soil showed no significant differences in Zn and Cr concentrations, while the contents of Cu, Pb, Cd, and Mn were decreased by 39.64%, 13.11%, 7.96%, and 4.64%, respectively (Fig. 3). Moreover, the temporal dynamics of individual heavy metal contents across different years exhibited no consistent pattern. In addition, the relative closeness of heavy metals in PaS soil decreased by 41.03%, with reductions of 49.37% and 33.33% in the 1st and 2nd years, respectively. Compared with Py soil, the relative closeness of heavy metals in PyS soil declined by 61.36%, showing decreases of 70.45% and 51.11% in the 1st and 2nd years, respectively (Fig. 4; Table S2).

Main-effect analysis showed that geographical location (η² = 0.33, *P* = 0.00) was the most dominant factor influencing the relative closeness of soil heavy metals, followed by planting pattern (η² = 0.31, *P* = 0.00) (Table S4). Meanwhile, year (η² = 0.01, *P* = 0.06) and season (η² = 0.001, *P* = 0.97) exerted no significant effects on the relative closeness of heavy metals. In addition, the interaction between geographical location and season had the most significant impact on the relative closeness of heavy metals (η² = 0.07, *P* = 0.00) (Table S4).

### Sensitivity analysis

**Fig. 5:**
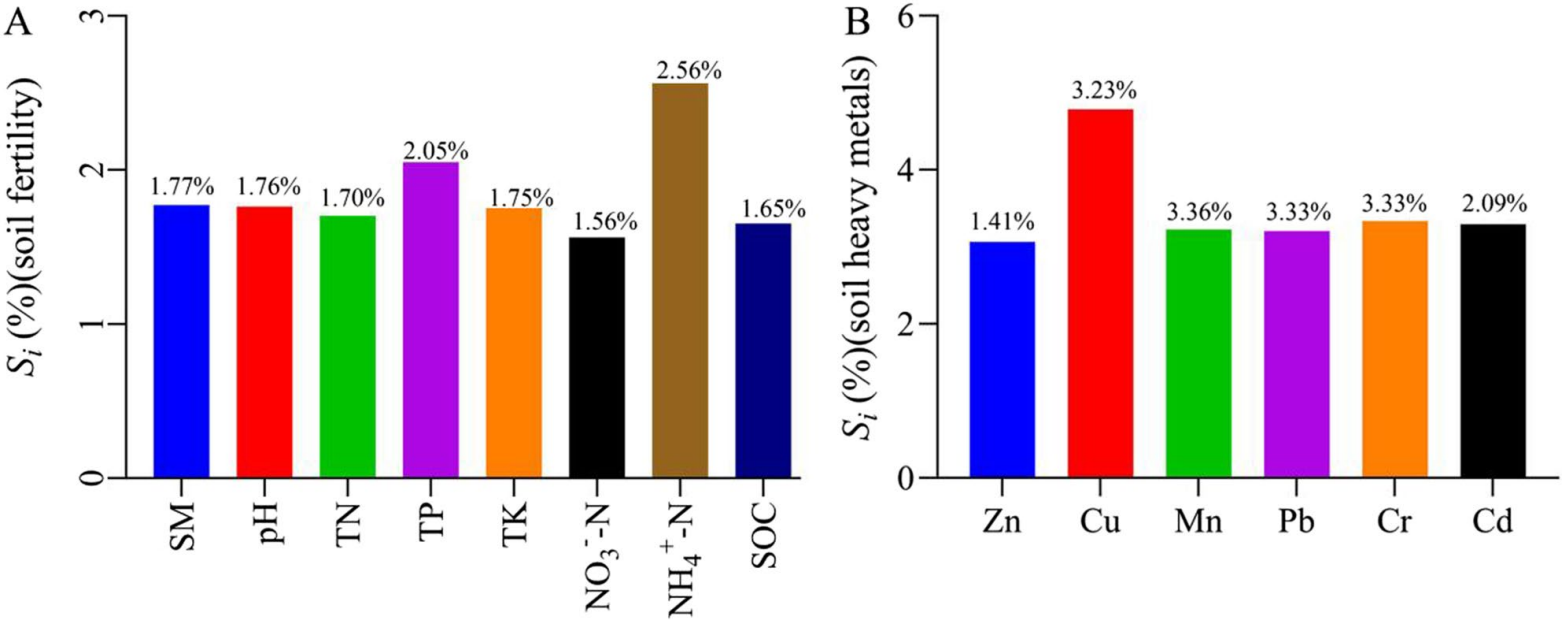
Sensitivity analysis of key indicators affecting soil fertility (**A**) and heavy metal dynamics (**B**)

NH_4_^+^–N and TP exhibited relatively high contributions to soil fertility, with sensitivity indices of 2.56% and 2.05%, respectively. By contrast, SWC, pH, TN, TK, NO_3_^–^–N, and SOC made relatively low contributions, with their sensitivity ranked as: SWC > pH > TK > TN > SOC > NO_3_^–^–N. Furthermore, Cu showed the most significant influence on heavy metal assessment, with a sensitivity index of 3.23%. The effects of the remaining heavy metals followed the order: Mn > Cr = Pb > Cd > Zn (Fig. 5).

### Analysis of Pi and NPi of soil heavy metals

Compared with Pa soil, the single-factor pollution index (Pi) values of Zn, Cu, Mn, Cr, and Cd in PaS soil decreased significantly, whereas no significant difference was observed in the Pi value of Pb. Relative to Py soil, only the Pi values of Cu and Cd in PyS soil declined significantly, while those of Zn, Mn, Pb, and Cr remained unchanged (Table S5). According to the classification criteria outlined in the Soil Environmental Quality Risk Control Standard for Agricultural Land (GB 15618 − 2018) (Table S6 and S7). The Pi values of all heavy metals across different systems were ≤ 1.0, meeting the GradeⅠ(clean/safe) standard.

Nemerow pollution index (NPi) revealed that heavy metals NPi in PaS soil decreased by 7.72% compared with Pa soil, with reductions of 8.35% and 7.02% in the 1st and 2nd years, respectively. Compared with Py soil, the NPi in PaS soil decreased by 2.31%, and the reduction was mainly observed in the second year (3.28%) (Table S5). Additionally, the pollution levels were all classified as GradeⅠ(clean/safe), indicating that the soils in all the systems were generally in a safe condition, with a sound ecological environment and free from heavy metal contamination.

### Abundance of AOA and AOB analysis

**Fig. 6:**
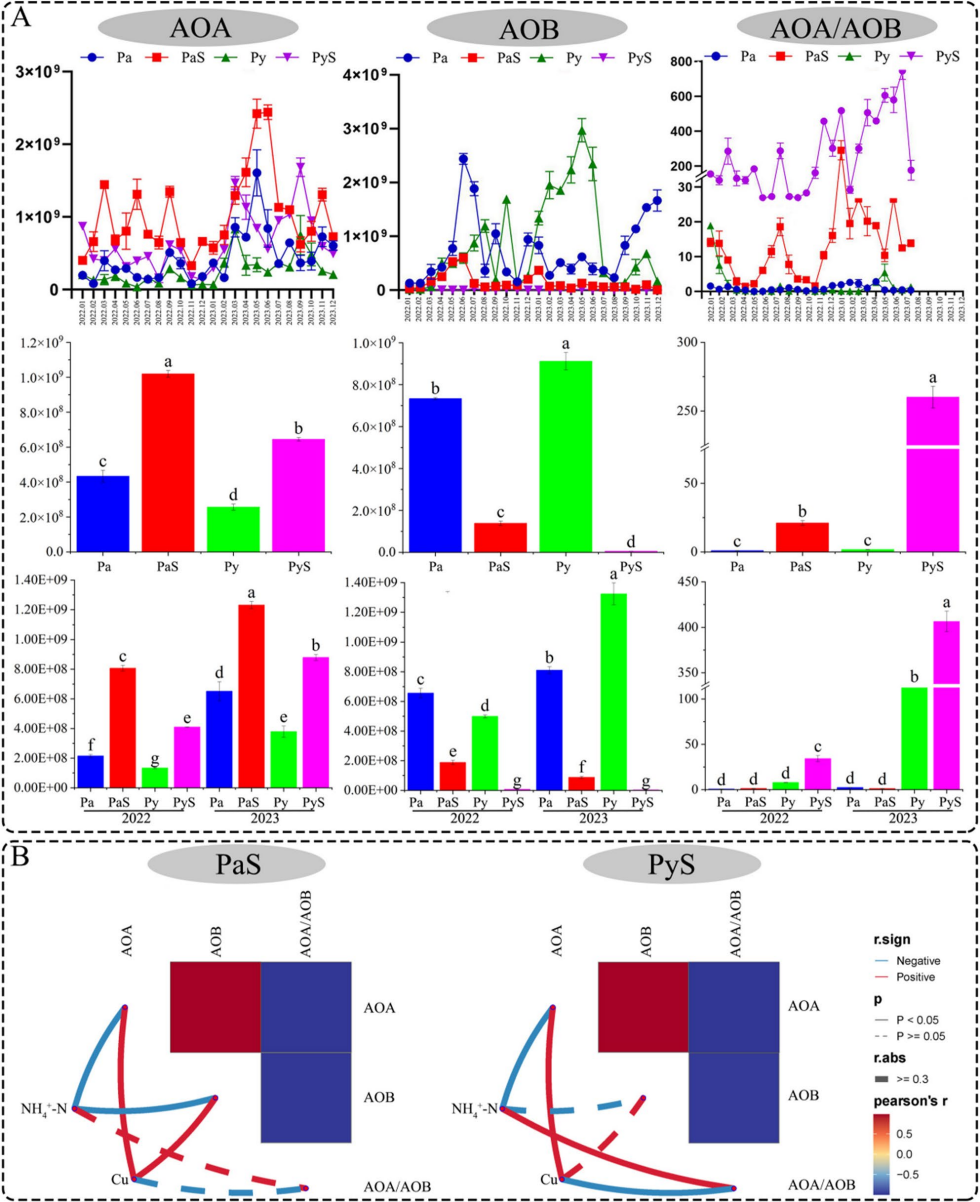
Temporal dynamics and interrelationships of AOA and AOB under different cropping systems. **A** Temporal variations in the abundance of AOA, AOB, and the AOA/AOB ratio from 2022 to 2023, and their mean abundance in 2022 and 2023. Different lowercase letters indicate significant differences among treatments (*P* < 0.05). **B** Pearson correlation analysis between NH_4_^+^–N, Cu and the abundance of AOA, AOB, and the AOA/AOB ratio in PaS and PyS intercropping systems. The line color and thickness represent the direction and magnitude of the correlation coefficient, respectively

The abundance ranges of AOA, AOB, and AOA/AOB ratio were determined to be 3.67 × 10^7^ ~ 2.44 × 10^9^, 1.72 × 10^5^ ~ 2.68 × 10^9^, and 0.05 ~ 746.66, respectively. Compared to the control, AOA abundance in PaS and PyS soils increased by 135.25% and 151.44%, whereas AOB abundance was significantly decreased by 81.16% and 99.43%, respectively. Correspondingly, the AOA/AOB ratio was substantially elevated by 2,195.07% and 15,515.59%, respectively (Fig. 6A, B).

With increasing cultivation years, AOA abundance showed a clear increasing trend across all four systems. In contrast, AOB abundance increased only in the Pa system, decreased significantly in the PaS system, and exhibited no significant difference in the PyS system. Furthermore, the AOA/AOB ratio displayed an upward trend in the PaS and PyS systems, yet remained stable in both the Pa and Py systems (Fig. 6C).

Correlation analysis revealed that in the PaS system, both AOA and AOB abundances were significantly negatively correlated with NH_4_^+^–N but positively correlated with Cu. In the PyS system, AOA abundance tended to be negatively and positively correlated with NH_4_^+^–N and Cu, while the AOA/AOB ratio positively and negatively correlated with NH_4_^+^–N and Cu (Fig. 6D).

### SEM analysis

**Fig. 7:**
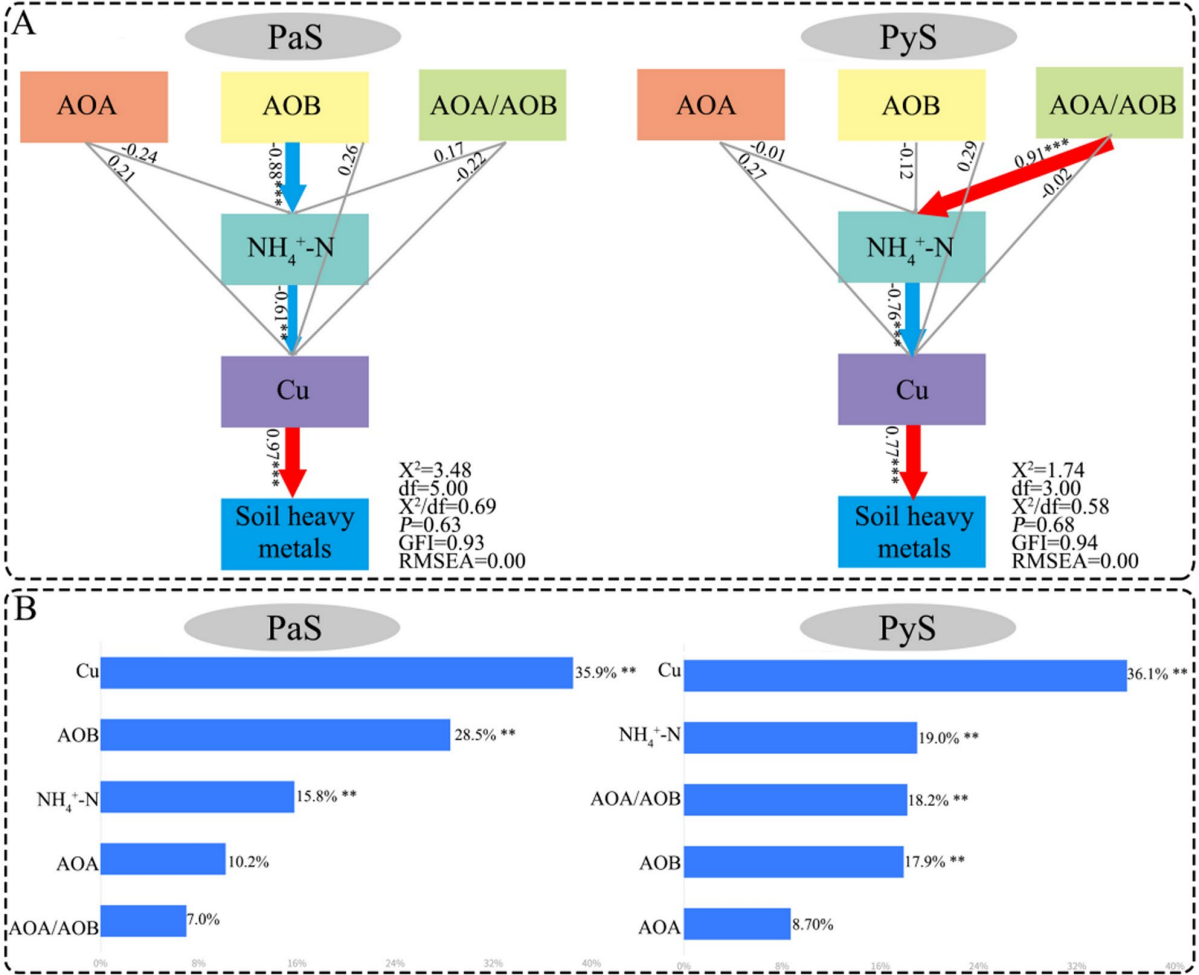
SEM (**A**) and random forest (**B**) analysis illustrate the regulatory pathways of ammonia-oxidizing microorganisms on soil heavy metal dynamics in intercropping systems. Red and blue arrows denote significant positive and negative effects, respectively, and gray arrows indicate non-significant paths. Significance levels: *P* < 0.05, **P < 0.01, ***P < 0.001

SEM results revealed significant differences in the regulatory pathways among ammonia-oxidizing microorganisms, NH_4_^+^–N, Cu, and soil heavy metals between the two intercropping systems (Fig. 7). In both PaS and PyS soils, AOB and AOA/AOB ratio exerted indirect effects on Cu accumulation, mediated by NH_4_^+^–N dynamics. Conversely, Cu demonstrated a strong direct effect on soil heavy metals (Fig. 7A; Table S8). These interactions established a differentiated cascading regulatory pathway characterized as “ammonia-oxidizing microorganisms → NH_4_^+^–N → Cu → total heavy metals”. Notably, the PaS system was primarily driven by AOB abundance, whereas the PyS system relied on the AOA/AOB ratio as the key variable regulating NH_4_^+^–N dynamics. Both models met the optimal fit criteria, confirming the existence of substantial microbial regulatory mechanisms underlying soil heavy metal accumulation in these conifer intercropping systems. Specifically, Cu content emerged as the most critical driver of heavy metal levels in both systems. In the PaS system, AOB and NH_4_^+^–N ranked as the second most significant drivers following Cu. In contrast, the PyS system exhibited comparable contributions from NH_4_^+^–N (19.0%), the AOA/AOB ratio (18.2%), and AOB (17.9%) (Fig. 7B).

## Discussion

### Mitigating effects of PaS and PyS intercropping systems on soil fertility, heavy metal accumulation, and pollution risks

Intercropping Sanqi with *P. armandii* and *P. yunnanensis* significantly increased soil NO_3_^−^−N and TP contents, while decreasing NH_4_^+^–N and TK contents. This observation is consistent with previous findings on soil element dynamics in Sanqi–*P. armandii* agroforestry systems [10]. The increase in TP is mainly attributed to phenolic acids secreted by Sanqi roots, which are capable of activating insoluble soil P and facilitating P release [36]. In contrast, the reduction in TK is associated with the continuous uptake and consumption of potassium by Sanqi during its growth period [37]. The simultaneous increase in NO_3_^−^−N and decrease in NH_4_^+^–N may result from the substantial mineralization of organic nitrogen released during organic matter decomposition. Specifically, organic nitrogen is rapidly converted to NH_4_^+^–N under the mediation of ammonia-oxidizing microorganisms, followed by further nitrification into NO_3_^−^−N [38]. TOPSIS analysis revealed that, compared with monoculture pine stands, the comprehensive soil fertility closeness degree was significantly improved by 19.35% and 18.18% in the PaS and PyS system, respectively. Such improvements stem from Sanqi cultivation practices—specifically tillage, organic fertilization, irrigation, and pine needle mulching—which synergistically enhance soil nutrient cycling [6, 39]. The PyS system showed stronger improvement in soil fertility relative to its local monoculture, while the PaS system also significantly enhanced soil fertility at its site. Previous studies have confirmed that soil NH_4_^+^–N content and its transformation rate directly determine soil nitrogen supply capacity and overall fertility level [40]. Similarly, our results indicate that NH_4_^+^–N acts as the most sensitive indicator reflecting changes in soil fertility.

Soil concentrations of Cu, Pb, Cd, and Mn exhibited a significant downward trend in the PaS and PyS systems. Notably, the PaS system further reduced Zn and Cr levels (Fig. 3). These findings differ from previous studies on Sanqi–*P. armandii* intercropping systems, which reported significant reductions specifically in Pb and Cr [41, 42]. Variations in temporal scale likely explain this discrepancy: previous studies focused on short-term (1-year) dynamics, whereas the current work systematically characterized heavy metal changes throughout Sanqi’s entire 2-year growth cycle. Furthermore, variations in heavy metals across different intercropping systems (e.g., *Pennisetum purpureum*–*Melia azedarach/Broussonetia papyrifera* and *Zea mays*–*Brassica juncea*) have been documented in existing literature [41, 42]. These differences are primarily ascribed to a complex interplay of multiple factors, including plant uptake rates [43], growth duration [42], soil texture [33], tillage practices [10], and planting density [44]. The entropy-weighted TOPSIS evaluation further indicated that the PaS and PyS systems reduced soil heavy metal relative closeness by 41.03% and 61.36%, respectively, with Cu identified as the most sensitive indicator affecting soil heavy metal status (Fig. 5). Sanqi appears to synergistically regulate soil heavy metals through multiple pathways. Sanqi root exudates, such as citric, oxalic, and amino acids, interact with heavy metal ions (e.g., Cd^2+^, Pb^2+^, Cu^2+^, Zn^2+^) via direct chelation [42], pH alteration [45], and microbial community shifts [19]. These interactions ultimately influence the solubility, mobility, and bioavailability of these metals [46]. Additionally, increased SOC content enhances the adsorption and passivation of heavy metal cations, thereby reducing their bioavailability and mobility [47]. Agronomic practices associated with Sanqi cultivation, including organic fertilizer application, tillage, and quicklime application, not only improve soil structure and adsorption capacity but also alter soil pH and redox potential, facilitating the transformation of heavy metals into stable forms. Notably, the PyS system exhibited superior heavy metal immobilization performance, which may be associated with the specific litter composition, root exudation patterns, and rhizosphere microbial characteristics of *P. yunnanensis* [5], collectively providing more favorable conditions for heavy metal stabilization.

In addition, a significant negative correlation was observed between soil fertility and heavy metal contents in both the PaS and PyS intercropping systems (Fig. 7), which is consistent with the findings of Liu et al. [10]. Main effect analysis demonstrated that geographical location (η² = 0.107, *P* = 0.000) was the primary factor determining soil fertility and heavy metal contents, followed by planting pattern (η² = 0.027, *P* < 0.01) (Table S3 and S4). Environmental factors inherent to geographical location, such as climatic characteristics, parent material, and topography, fundamentally determine soil fertility level and heavy metal background values [14]. In contrast, as an artificial regulation measure, planting pattern exerts a significant regulatory effect on the coupling relationship between soil fertility and heavy metals through various pathways, such as planting duration [48], plant species [49], root exudates [36], field management practices [50], and pine needle litter [51]. Previous studies have confirmed that the PaS system can not only enhance the heavy metal absorption capacity of Sanqi and pine trees [52] but also has no negative impacts on Sanqi quality [53] or pine tree growth [39]. Regarding the Nemerow pollution index, both the PyS and PaS systems showed a decreasing trend, with the PyS system showing a lower index value. Nevertheless, the soil pollution levels under the four land use patterns were all rated as Grade Ⅰ (clean/safe), indicating that the soil was generally in a safe state with a good ecological environment and no threat of heavy metal pollution (Table S7). The study soils were non-polluted natural forest soils with low heavy metal backgrounds; thus, observed reductions indicate metal immobilization and stabilization rather than phytoremediation. With Mn and Cr present at relatively high levels, future remediation of Sanqi soils should leverage novel technologies, including gene editing and synthetic biology, to ensure soil health [54].

### Mediating roles of ammonia-oxidizing microorganisms in nitrogen transformation and heavy metal regulation

Subtropical pine forests in Yunnan Province generally face N limitation [55], a condition that is exacerbated in Sanqi–pine agroforestry systems. This intensification is primarily attributed to the dual effects of increased N consumption by the understory crop and the prohibition of synthetic N fertilizers under organic management regimes [56]. As key drivers of nitrification, ammonia-oxidizing microorganisms not only directly determine N transformation and availability [18] but also indirectly regulate the mobility of heavy metals [19]. Consequently, they play an indispensable role in alleviating N limitation and improving the soil environment in the Sanqi–pine system.

Compared with the Pa and Py systems, the abundance of AOA in the PaS and PyS systems was significantly increased by 135.25% and 151.44%, respectively. In contrast, the abundance of AOB was significantly decreased by 81.16% and 99.43%, respectively. This led to a significant increase in the AOA/AOB ratio, indicating a distinct community succession toward AOA dominance. This finding aligns with previous studies suggesting that management practices and environmental disturbances are potent drivers of ammonia-oxidizing community structure [57]. Ecologically, AOA are generally considered to possess a competitive advantage over AOB in oligotrophic, acidic, or extreme environments due to their higher substrate affinity, whereas AOB tend to thrive in environments with higher ammonia concentrations [6, 21]. Furthermore, heavy metals exert toxic stress on microbial communities; yet AOA, possessing core functional genes (amoA) and intrinsic heavy metal tolerance genes, exhibit stronger adaptability to metal-stressed than AOB [23, 24]. Our results showed that AOA abundance was negatively and positively correlated with NH_4_^+^–N and Cu, suggesting a tight coupling between ammonia-oxidizing microorganisms, nitrogen transformation, and heavy metal dynamics. Mechanistically, NH_4_^+^–N transformation reduces Cu mobility and bioavailability through integrated geochemical and biochemical processes. NH_4_^+^ competes with Cu^2+^ for soil colloid adsorption sites, while ammonia oxidation modulates soil pH to regulate Cu precipitation and mineral adsorption. Enhanced nitrogen cycling also accumulates organic ligands that form stable complexes with Cu. Meanwhile, altered AOA/AOB community structure reshapes the rhizosphere microenvironment, further facilitating Cu immobilization and reducing its migration potential [32, 33]. Hence, the extreme reduction in AOB and sharp rise in AOA/AOB ratio represent an important adaptive strategy of the microbial community under intercropping. This shift enhances NH_4_^+^–N transformation, promotes Cu immobilization, and reduces heavy metal mobility, thereby stabilizing soil N cycling and reducing heavy metal risks. Collectively, these soil physicochemical factors (N and Cu) likely serve as the key environmental filters driving the differential succession of AOA and AOB communities.

SEM showed that AOB in PaS system and the AOA/AOB ratio in PyS system indirectly influenced Cu content by modulating NH_4_^+^–N transformation, while Cu directly drove the accumulation of total heavy metals, forming an “ammonia-oxidizing microorganisms–NH_4_^+^–N–Cu–heavy metals” cascade pathway (Fig. 7A, B). This difference suggests that different Sanqi intercropping systems have formed unique microbial functional pathways, thereby regulating soil N cycling and heavy metal form transformation. Overall, this adaptive transformation of the ammonia-oxidizing microbial community structure in the Sanqi-pine agroforestry system is an important ecological response mechanism of the system under the dual pressures of nitrogen deficiency and heavy metal stress, which is of positive significance for maintaining the balance of system nitrogen cycling and improving soil heavy metal pollution. Building on this, regulating the AOA/AOB ratio through agronomic practices (e.g., organic amendment, litter management) is expected to improve soil nitrogen use efficiency and heavy metal immobilization capacity, providing a new effective microbial-based method for soil health regulation in the Sanqi-pine agroforestry system. Additionally, drought, extreme climate events, and different fertilization regimes can interact with microbial-mediated heavy metal regulation. Future work will focus on developing single-cell, spatial transcriptomics, and metagenomics [6, 58, 59] to elucidate the heavy metal regulation of Sanqi plants and ammonia-oxidizing microorganisms under these environments. Further controlled experiments are needed to clarify their interactive relationships under different conditions.

## Conclusions

This 24-month study investigated soil fertility, heavy metal dynamics, and ammonia-oxidizing microorganisms in *P. armandii* (Pa), *P. yunnanensis* (Py) monocultures and their Sanqi intercropping systems (PaS, PyS). Results demonstrated that both PaS and PyS systems effectively improved soil fertility and reduced heavy metal risks, with the PyS system exhibiting superior comprehensive performance at its experimental site. Sensitivity analysis identified NH_4_^+^–N and Cu as the most sensitive indicators for soil fertility and heavy metal assessment, respectively. Sanqi intercropping significantly elevated AOA abundance and the AOA/AOB ratio while suppressing AOB, indicating a community shift toward AOA dominance under oligotrophic and heavy metal stress. SEM verified a cascading regulatory pathway: ammonia-oxidizing microorganisms → NH_4_^+^–N → Cu → total heavy metals, with AOB as the dominant driver in the PaS system and the AOA/AOB ratio as the core regulator in the PyS system. Overall, our results offer a site-specific scientific basis for constructing ecologically safe cultivation modes of Sanqi, supporting the coordinated development of medicinal plant quality, soil environmental safety, and sustainable forest management. Geographical location dominates soil properties, and direct cross-site comparison between PaS and PyS is limited. Future work should include multi-site monitoring and multi-omics to identify key functional genes and metabolic pathways of AOA/AOB mediating heavy metal immobilization.

## Supplementary Information

Supplementary Material 1.

## Data Availability

All data supporting the findings of this study are available within the paper and its Supplementary Information.

## References

[1] Hei JY, Li Y, Rui R, He S, Wang B, Wang S. Rare microbial α-diversity and network complexity significantly enhance soil multifunctionality in Pinus armandii under Sanqi-pine agroforestry system. Ind Crop Prod. 2026;243:123108.

[2] Wang CY, Mao GM, Li YB, Zi WJ, Wang QY, Huang HC. Tree root-mediated soil metabolome in agroforestry enhancing the growth and quality of Panax notoginseng. Plant Soil. 2025;507:497–518.

[3] Chen SY, Rui R, Wang S, He XH. Comparative analysis of the floral fragrance compounds of *Panax notoginseng* flowers under the *Panax notoginseng*-Pinus agroforestry system using SPME-GC-MS. Molecules. 2022;27:3565. 35684502 10.3390/molecules27113565 PMC9182305

[4] Hei JY, Li Y, Wang Q, Wang S, He XH. Effects of exogenous organic acids on the soil metabolites and microbial communities of *Panax notoginseng* from the forest understory. Agronomy. 2024;14:601.

[5] Hei JY, Li Y, Rui R, Faisal N, Peng JS, Wang B, et al. The cultivation of *Panax notoginseng* enhances the metabolites and microbial network complexity in the soil of *Pinus armandii* rather than *Pinus kesiya*. Front Microbiol. 2025;16:1616266. 40842843 10.3389/fmicb.2025.1616266 PMC12364910

[6] He S, Rui R, Hei JY, Li Y, Faisal N, Wang B, et al. Organically managed Sanqi alters the soil C metabolism and purine metabolism pathway through metagenomic and metabolomic analyses. Plant Soil. 2025;25:1–7.

[7] Shen FY, Fei LJ, Tuo YF, Peng YL, Yang QL, Zheng RQ, et al. Effects of water and fertilizer regulation on soil physicochemical properties, bacterial diversity and community structure of *Panax notoginseng*. Sci Hort. 2024;326:112777.

[8] Tuo YF, Luo XQ, Wang ZY, Liang JP, Shi R, Wang ZX, et al. Effects of water and fertilizer regulation on soil microbial community, fruit nutrients, and saponin content of *Panax notoginseng*: A three years field experiment. Ind Crop Prod. 2024;220:119166.

[9] Wan YN, Liu J, Zhuang Z, Wang Q, Li HF. Heavy metals in agricultural soils: sources, influencing factors, and remediation strategies. Toxics. 2024;12(1):63. 38251018 10.3390/toxics12010063 PMC10819638

[10] Liu KY, Zhao XY, Rui R, Li Y, Hei JY, Yu LF, et al. Effects of sanqi cultivation on soil fertility and heavy metal content in the sanqi–pine agroforestry system. Agronomy. 2025;15:2123.

[11] Milosavljevic JS, Serbula SM, Cokesa DM, Milanovic DB, Radojevic AA, Kalinovic TS, et al. Soil enzyme activities under the impact of long-term pollution from mining-metallurgical copper production. Eur J Soil Biol. 2020;101:103232.

[12] Gui RY, Hu YY, Li Q, Zhuang SY. Effect of cultivation time on soil heavy metal accumulation and bioavailability in. Pedosphere. 2020;30(6):810–6.

[13] Gong B, Wang XP, Ying RR, Qiu H. Impact of soil moisture on the bioavailability and ecotoxicity of heavy metals. Asian J Ecotoxicol. 2025;20(01):48–61.

[14] Wang JL, Dong CY, Sun SJ, Peng SQ, Mu LY, Zhang NM. Characteristics and risk assessment of heavy metal contamination in arable soils developed from different parent materials. Agriculture. 2024;14(11):2010.

[15] Jiang YF, Ye YC, Guo X. Spatiotemporal variation of soil heavy metals in farmland influenced by human activities in the Poyang Lake region, China. CATENA. 2019;176:279–88.

[16] Gan T, Zhao HW, Ai Y, Zhang SH, Wen YL, Tian LM. Spatial distribution and ecological risk assessment of heavy metals in alpine grasslands of the Zoige Basin, China. Front Ecol Evol. 2023;11:1093823.

[17] Sinha S, Basak A, Mondol MSA. Understanding heavy metal accumulation in crops: sources, plant responses, tolerance mechanisms, and environmental effects. J Environ Sci Health C. 2025;43(3):269–94. 10.1080/26896583.2025.2521207 40579819

[18] Liang D, Ouyang Y, Tiemann L, Robertson GP. Niche differentiation of bacterial versus archaeal soil nitrifiers induced by ammonium inhibition along a management gradient. Front Microbiol. 2020;11. 10.3389/fmicb.2020.568588 PMC7689314 33281763

[19] Zhou C, Gao Y, Ma Q, Xia Z, Zhu M, Zhang X, et al. The single and combined effects of sulfamethazine and cadmium on soil nitrification and ammonia-oxidizing microorganisms. Environ Sci Pollut R. 2023;30(19):56108–20. 10.1007/s11356-023-26141-y 36913014

[20] Huang X, Li X, Zheng L, Zhang Y, Sun L, Feng Y, et al. Comprehensive assessment of health and ecological risk of cadmium in agricultural soils across China: A tiered framework. J Hazard Mater. 2024;465:133111. 38043426 10.1016/j.jhazmat.2023.133111

[21] Liu XJ, Shao YF, Dong YP, Dong MY, Xu ZW, Hu XX, et al. Response of ammonia-oxidizing archaea and bacteria to sulfadiazine and copper and their interaction in black soils. Environ Scie Pollut Res. 2020;28(9):11357–68. 10.1007/s11356-020-11356-0 33123879

[22] He H, Dang L, Yang Q, Chen R, Yang JM, Li JS, et al. Cadmium toxicity on communities of ammonia-oxidizing microorganisms. Peer J. 2025;13:18829. 10.7717/peerj.18829 PMC11849506 39995999

[23] Li MY, Zhang JC, Yang X, Zhou YY, Zhang LH, Yang Y, et al. Responses of ammonia-oxidizing microorganisms to biochar and compost amendments of heavy metals-polluted soil. J Environ Sci. 2021;102:263–72. 10.1016/j.jes.2020.09.029 33637252

[24] Liu JL, Li C, Ma WD, Wu ZX, Liu W, Wu WX. Exploitation alters microbial community and its co-occurrence patterns in ionic rare earth mining sites. Sci Total Environ. 2023;898:165532. 37454857 10.1016/j.scitotenv.2023.165532

[25] Chen YG, He XLS, Huang JH, Luo R, Ge HZ, Wołowicz A. Impacts of heavy metals and medicinal crops on ecological systems, environmental pollution, cultivation, and production processes in China. Ecotox Environ Safe. 2021;219:112336. 10.1016/j.ecoenv.2021.112336 34044310

[26] Bao H, Wang Y, Bao H, Wang F, Jiang Q, He X. Ecotoxicological impacts of heavy metals on medicinal plant quality and rhizosphere microbial communities. Plants. 2025;14(20):3214. 41157771 10.3390/plants14203214 PMC12566869

[27] Liao J, Xia P. Continuous cropping obstacles of medicinal plants: Focus on the plant-soil-microbe interaction system in the rhizosphere. Sci Hortic. 2024;328:112927.

[28] Yang L. Factors that affect the form of heavy metals in traditional Chinese medicines planting environment. J Anhui Agricultural Sci. 2013;41(24):9940–2.

[29] Asgari Lajayer B, Ghorbanpour M, Nikabadi S. Heavy metals in contaminated environment: Destiny of secondary metabolite biosynthesis, oxidative status and phytoextraction in medicinal plants. Ecotox Environ Safe. 2017;145:377–90. 10.1016/j.ecoenv.2017.07.035 28759767

[30] Abdelkrim S, Jebara SH, Saadani O, Abid G, Taamalli W, Zemni H. In situ effects of Lathyrus sativus- PGPR to remediate and restore quality and fertility of Pb and Cd polluted soils. Ecotox Environ Safe. 2020;192:110260. 10.1016/j.ecoenv.2020.110260 32050135

[31] Asiminicesei DM, Fertu DI, Gavrilescu M. Impact of heavy metal pollution in the environment on the metabolic profile of medicinal plants and their therapeutic potential. Plants. 2024;13(6):913. 38592933 10.3390/plants13060913 PMC10976221

[32] Li P, Hao H, Zhang Z, Mao X, Xu J, Lv Y, et al. A field study to estimate heavy metal concentrations in a soil-rice system: Application of graph neural networks. Sci Total Environ. 2022;832:155099. 35398437 10.1016/j.scitotenv.2022.155099

[33] Huang X, Tang S, Zeng M, Qin Z, Liang J, Chen Y, et al. Soil aggregate size mediates the variations in the abundance and function of ammonia oxidizers in heavy metal-contaminated soil under different nitrogen fertilization regimes. Appl Soil Ecol. 2024;200:105448.

[34] Wang YF, Gu JD, Dick RP, Han W, Yang HX, Liao HQ, et al. Distribution of ammonia-oxidizing archaea and bacteria along an engineered coastal ecosystem in subtropical China. Ecotoxicology. 2021;30(8):1769–79. 33432457 10.1007/s10646-020-02327-9

[35] Cao XX, Zhao W, Zhang H, Lin J, Hu JY, Lou YH., et al. Individual and combined contamination of oxytetracycline and cadmium inhibited nitrification by inhibiting ammonia oxidizers. Front Microbiol. 2022;13:1062703. 10.3389/fmicb.2022.1062703 PMC9751337 36532490

[36] Wang W, Chen Y, Zhang F, Zhang W, Liu J, Wang J. Cotton-maize intercropping increases rhizosphere soil phosphorus bioavailability by regulating key phosphorus cycling genes in northwest China. Appl Soil Ecol. 2023;182:104734.

[37] Sun B, Pan XZ, Wang JD, Han XZ, Zhang YM, Hai MD, et al. Effect of nutrient balance on spatial and temporal change of soil fertility in different agriculture area in China. Adv Earth Sci. 2008;23(11):8.

[38] Guo W, Zhang Z, Liu Q, Xiao J, Yin H. Seasonal variations in plant nitrogen acquisition in an ectomycorrhizal alpine forest on the eastern Tibetan Plateau, China. Plant Soil. 2020;459:79–91.

[39] Rui R, Hei JY, Li Y, Wan XL, Wang S, He XH. Alleviating microbial carbon limitation in *Pinus armandii* forests through *Panax notoginseng* cultivation. Forests. 2025;16:158.

[40] Zhang Q, Chen M, Leng Y, Wang X, Fu Y, Wang D. Organic substitution stimulates ammonia oxidation-driven N. Sci Total Environ. 2023;872:162183. 36804975 10.1016/j.scitotenv.2023.162183

[41] Wang XH, Xiao XY, Guo ZH, Peng C, Wang XY. Potential of intercropping pennisetum purpureum schum with melia azedarach L. and broussonetia papyrifera for phytoremediation of heavy-metal contaminated soil around mining areas. Environ Sci. 2023;44(1):426–35. 10.13227/j.hjkx.202203259 36635830

[42] Chi G, Fang Y, Zhu B, Guo N, Chen X. Intercropping with. J Hazard Mater. 2024;461:132727. 37813037 10.1016/j.jhazmat.2023.132727

[43] Liu ZQ, Yang JG, Wu ZH, Li ZF, Song BL, Feng RW. Factors restraining uptake/translocation of heavy metals (metalloids) related with plant roots and its mechanisms. Res Agricultural Modernization. 2021;42(2):284–93.

[44] Yang Q, Wang XH, Ao HJ, Luo XR, Yang L, Liu QF. Remediation effect on heavy metal pollution of sorghum planting density. J Hunan Agricultural Univ. 2018;44(03):234–9.

[45] Narayanan M, Ma Y. Mitigation of heavy metal stress in the soil through optimized interaction between plants and microbes. J Environ Manage. 2023;345:118732. 10.1016/j.jenvman.2023.118732 37536126

[46] Wang Y, Feng FY, Ge J, Li Y, Yu XY. Effects and mechanisms of plant root exudates on soil remediation. Acta Ecol Sinica. 2022;42(3):829–42.

[47] Khaledian Y, Pereira P, Brevik EC, Pundyte N, Paliulis D. The influence of organic carbon and pH on heavy metals, potassium, and magnesium levels in Lithuanian podzols. Land Degrad Dev. 2016;28(1):345–54.

[48] Xiong C, Wang R, Dou X, Luo C, Wang X, Xiao W. Soil moisture, nutrients, root distribution, and crop combination benefits at different water and fertilizer levels during the crop replacement period in an apple intercropping system. Agronomy. 2023;13(11):2706.

[49] Nasar J, Ahmad M, Gitari H, Tang L, Chen Y, Zhou XB. Maize/soybean intercropping increases nutrient uptake, crop yield and modifies soil physio-chemical characteristics and enzymatic activities in the subtropical humid region based in Southwest China. BMC Plant Biol. 2024;24(1):434. 38773357 10.1186/s12870-024-05061-0 PMC11106902

[50] Wen J, Upchurch R, Zak DR. Ammonium oxidation by bacteria and archaea have functional implications for nitrification across a forested landscape. Ecosphere. 2024;15(12):4958.

[51] Zhang L, Shen Y, Hu Y, Li J, Liu Y, Chen S. Response of soil phosphorus fractions to litter removal in subalpine coniferous forest. Sci Total Environ. 2023;898:166383. 37598961 10.1016/j.scitotenv.2023.166383

[52] Yang RG, Wang ML, Rui R, Zhao XY, Zhang XY, Xiong BJ. Distribution characteristics of mineral elements and pollution evaluation of heavy metals in pinus armandii-panax notoginseng planting pattern. J Shandong Agric Sci. 2023;55:127–33.

[53] Li Y, Hei JY, Wang B, Wang S, He XH. Unraveling the molecular mechanisms of flavonoid biosynthesis in *Panax notoginseng* flowers across planting patterns and developmental stages using integrated metabolomics and transcriptomics analyses. Sci Hortic. 2024;335:9.

[54] Ali I, White JC, Ali M. Transforming toxic element remediation: a necessary path for 21st-century food security. Trends Plant Sci. 2026;31(5):514–7. 41139567 10.1016/j.tplants.2025.10.008

[55] Nian Y, Chen W, Zhao Y, Hou Z, Zhang L, Liang X, Song. Yet al. Response of litter decomposition and nutrient release characteristics to simulated N deposition in pinus yunnanensis franch. Forest in Central Yunnan Plateau. Forests. 2025;16(4):684.

[56] Zhao XY, He S, Rui R, Hei JY, He XH, Wang S. Introduction of Panax notoginseng into pine forests significantly enhances the diversity, stochastic processes, and network complexity of nitrogen-fixing bacteria in the soil. Front Microbiol. 2025;16:1531875. 39963494 10.3389/fmicb.2025.1531875 PMC11830724

[57] Cai FF, Luo Y, Yang JF, Irfan M, Zhang SY, An N, et al. Effect of long-term fertilization on ammonia-oxidizing microorganisms and nitrification in brown soil of northeast China. Front Microbiol. 2021;11:622454. 33613469 10.3389/fmicb.2020.622454 PMC7890093

[58] Ali M, Wang ZJ, Guo QY, Wang YX, Cai Y, Du J, et al. Mapping plant cell-type-specific responses to environmental stresses. Trends Plant Sci. 2026;1:1–27. 10.1016/j.tplants.2026.03.003 41956877

[59] Li Y, Hei JY, Peng JS, Zhao XY, Rui R, Wang B, et al. Unveiling the molecular regulations of terpenoid biosynthesis in Sanqi flowers across two cultivation systems and flowering stages using multiomics approaches. Ind Crops Prod. 2025;222:122074.

