# Supplementary material for: Coupled regulation effects of sanqi-pine intercropping systems on soil fertility and heavy metals mediated by ammonia-oxidizing microorganisms
Source: BMC Plant Biol. 2026 May 20;26:1190. doi: 10.1186/s12870-026-09050-3 (PMC13366968; doi:10.1186/s12870-026-09050-3)
Supplement: Supplementary file 1 — Supplementary Material 1. [file 12870_2026_9050_MOESM1_ESM.docx]

**Coupled regulation effects of sanqi-pine intercropping systems on soil fertility and heavy metals mediated by ammonia-oxidizing microorganisms**

**Jingying Hei ^a^, Qianqian Dong ^a^, Yingjun Li ^a^, Rui Rui ^b*^, Xiahong He ^b*^, Shu Wang ^a*^**

^a^ Yunnan Key Laboratory of Landscape Plant Resource Cultivation and Application, Southwest Forestry University, Kunming 650224, China

^b^ Yunnan Provincial Key Laboratory for Conservation and Utilization of In-forest Resources, Southwest Forestry University, Kunming 650224, China

^*^ Corresponding authors. E-mail addresses: [ruirui@swfu.edu.cn (R. Rui);](mailto:Ruirui@swfu.edu.cn;) hxh@swfu.edu.cn (X. He); wangshu@swfu.edu.cn (S. Wang).

**Table S1.** The primers sequences of AOA and AOB.

| **Target region** | **Primers** | **Sequences** |
| --- | --- | --- |
| AOA | AmoAF  amoAR | 5'-STAATGGTCTGGCTTAGACG-3'  5'-GCGGCCATCCATCTGTATGT-3' |
| AOB | bamoA1F  bamoA2R | 5'-GGGGTTTCTACTGGTGGT-3'  5'-CCCCTCKGSAAAGCCTTCTTC-3' |

**Table S2.** Average relative closeness of soil fertility and heavy metals.

|  | **Soil fertility** | **Soil heavy metals** |
| --- | --- | --- |
| Pa | 0.31±0.01^d^ | 0.78 ± 0.01^a^ |
| PaS | 0.37±0.00^c^ | 0.46±0.02^b^ |
| Py | 0.44±0.01^b^ | 0.44±0.03^b^ |
| PyS | 0.52±0.01^a^ | 0.17±0.03^c^ |

**Table S3.** Effects of geographical location, year, season, planting pattern, and their interactions on relative soil fertility closeness.

| **Factors** | **df** | **SS** | **F** | ***P*** | **η2** |
| --- | --- | --- | --- | --- | --- |
| Geographical location | 1 | 1.51 | 30.54 | 0 | 0.107 |
| Year | 1 | 0.00 | 0.06 | 0.809 | 0.000 |
| Seasonal dynamics | 3 | 0.00 | 0.04 | 0.991 | 0.000 |
| Planting pattern | 1 | 0.36 | 7.20 | 0.008 | 0.027 |
| Geographical location×Year | 1 | 0.87 | 17.71 | 0 | 0.065 |
| Geographical location×Seasonal dynamics | 3 | 1.30 | 26.24 | 0 | 0.235 |
| Geographical location×Planting pattern | 1 | 0.01 | 0.20 | 0.653 | 0.001 |
| Year×Seasonal dynamics | 3 | 0.05 | 1.06 | 0.368 | 0.012 |
| Year×Planting pattern | 1 | 0.45 | 9.11 | 0.003 | 0.034 |
| Seasonal dynamics×Planting pattern | 3 | 0.12 | 2.39 | 0.069 | 0.027 |
| Geographical location×Year×Seasonal dynamics | 3 | 0.30 | 6.13 | 0 | 0.067 |
| Geographical location×Year×Planting pattern | 1 | 0.15 | 3.10 | 0.079 | 0.012 |
| Geographical location×Seasonal dynamics×Planting pattern | 3 | 0.61 | 12.35 | 0 | 0.126 |
| Year×Seasonal dynamics×Planting pattern | 3 | 0.44 | 8.95 | 0 | 0.095 |
| Geographical location×Year×Seasonal dynamics×Planting pattern | 3 | 0.31 | 6.26 | 0 | 0.068 |

Note: η² = partial eta-squared (η_p_²). High-order three-way and four-way interactions were interpreted cautiously due to low degrees of freedom and limited statistical power.

**Table S4.** Effects of geographical location, year, season, planting pattern, and their interactions on relative soil heavy metal closeness.

| **Factors** | **df** | **SS** | **F** | ***P*** | **η2** |
| --- | --- | --- | --- | --- | --- |
| Geographical location | 1 | 7.06 | 126.36 | 0 | 0.33 |
| Year | 1 | 0.20 | 3.62 | 0.058 | 0.014 |
| Seasonal dynamics | 3 | 0.00 | 0.08 | 0.973 | 0.001 |
| Planting pattern | 1 | 6.42 | 114.91 | 0 | 0.31 |
| Geographical location×Year | 1 | 0.00 | 0.02 | 0.903 | 0 |
| Geographical location×Seasonal dynamics | 3 | 0.38 | 6.85 | 0 | 0.074 |
| Geographical location×Planting pattern | 1 | 0.06 | 1.03 | 0.312 | 0.004 |
| Year×Seasonal dynamics | 3 | 0.01 | 0.12 | 0.952 | 0.001 |
| Year×Planting pattern | 1 | 0.18 | 3.18 | 0.076 | 0.012 |
| Seasonal dynamics×Planting pattern | 3 | 0.13 | 2.27 | 0.081 | 0.026 |
| Geographical location×Year×Seasonal dynamics | 3 | 0.23 | 4.12 | 0.007 | 0.046 |
| Geographical location×Year×Planting pattern | 1 | 0.01 | 0.16 | 0.688 | 0.001 |
| Geographical location×Seasonal dynamics×Planting pattern | 3 | 0.02 | 0.28 | 0.841 | 0.003 |
| Year×Seasonal dynamics×Planting pattern | 3 | 0.19 | 3.40 | 0.018 | 0.038 |
| Geographical location×Year×Seasonal dynamics×Planting pattern | 3 | 0.01 | 0.13 | 0.942 | 0.002 |

Note: η² = partial eta-squared (η_p_²). High-order three-way and four-way interactions were interpreted cautiously due to low degrees of freedom and limited statistical power.

**Table S5.** Single-factor pollution and Nemerow pollution index of six heavy metals at different years.

|  |  | ***P_i_* (Zn)** | ***P_i_* (Cu)** | ***P_i_* (Mn)** | ***P_i_* (Pb)** | ***P_i_* (Cr)** | ***P_i_* (Cd)** | ***P_com_*** |
| --- | --- | --- | --- | --- | --- | --- | --- | --- |
| 1st  (2022) | Pa | 0.42 ± 0.01^a^ | 0.54 ± 0.01^b^ | 0.54 ± 0.01^a^ | 0.79 ± 0.01^a^ | 0.76 ± 0.00^a^ | 0.49 ± 0.00^a^ | 0.70 ± 0.00^a^ |
|  | PaS | 0.33 ± 0.01^c^ | 0.33 ± 0.00^c^ | 0.31 ± 0.01^e^ | 0.71 ± 0.00^b^ | 0.74 ± 0.00^a^ | 0.47 ± 0.00^c^ | 0.64 ± 0.00^b^ |
|  | Py | 0.18 ± 0.00^d^ | 0.09 ± 0.00^d^ | 0.42 ± 0.00^b^ | 0.38 ± 0.00^f^ | 0.70 ± 0.00^c^ | 0.49 ± 0.00^a^ | 0.56 ± 0.00^f^ |
|  | PyS | 0.17 ± 0.00^d^ | 0.05 ± 0.00^e^ | 0.37 ± 0.00^d^ | 0.29 ± 0.01^g^ | 0.71 ± 0.01^b^ | 0.45 ± 0.00^d^ | 0.56 ± 0.00^f^ |
| 2nd  (2023) | Pa | 0.39 ± 0.02^b^ | 0.60 ± 0.00^a^ | 0.37 ± 0.00^d^ | 0.40 ± 0.00^e^ | 0.76 ± 0.00^a^ | 0.48 ± 0.00^b^ | 0.65 ± 0.00^b^ |
|  | PaS | 0.33 ± 0.01^c^ | 0.33 ± 0.00^c^ | 0.43 ± 0.01^b^ | 0.40 ± 0.00^e^ | 0.72 ± 0.00^b^ | 0.47 ± 0.00^c^ | 0.60 ± 0.00^d^ |
|  | Py | 0.14 ± 0.00^e^ | 0.06 ± 0.00^e^ | 0.37 ± 0.00^d^ | 0.63 ± 0.01^c^ | 0.72 ± 0.01^b^ | 0.48 ± 0.00^b^ | 0.61 ± 0.00^c^ |
|  | PyS | 0.16 ± 0.00^d^ | 0.04 ± 0.00^f^ | 0.39 ± 0.00^c^ | 0.59± 0.00^d^ | 0.69 ± 0.02^c^ | 0.44 ± 0.00^e^ | 0.59 ± 0.01^e^ |

**Table S6.** Risk screening values for heavy metals in soil environmental quality.

| **Heavy Metals** | **Risk Screening Value (mg/kg)** | | | |
| --- | --- | --- | --- | --- |
| Zn | 200 | 200 | 250 | 300 |
| Cu | 50 | 50 | 100 | 100 |
| Mn | 626 | 626 | 626 | 626 |
| Pb | 70 | 90 | 120 | 170 |
| Cr | 150 | 150 | 200 | 250 |
| Cd | 0.3 | 0.3 | 0.3 | 0.6 |
| pH | pH≤5.5 | 5.5<pH≤6.5 | 6.5<pH≤7.5 | pH > 7.5 |

**Table S7.** Classification criteria for single-factor pollution Index and Nemerow pollution index (Li et al., 2021).

| **Pollution Class** | ***P_i_*** | **Pollution Degree** | ***P_Com_*** | **Pollution Degree** |
| --- | --- | --- | --- | --- |
| Ⅰ | *P_i_* ≤ 1.0 | Safety | *P_Com_* ≤ 0.7 | Clean (safety) |
| Ⅱ | 1.0 < *P_i_* ≤ 2.0 | Slight pollution | 0.7 < *P_Com_* ≤ 1.0 | Clean (threshold) |
| Ⅲ | 2.0 < *P_i_* ≤ 3.0 | Moderate pollution | 1.0 < *P_Com_* ≤ 2.0 | Slight pollution |
| Ⅳ | 3.0 < *P_i_* | Strong pollution | 2.0 < *P_Com_* ≤ 3.0 | Moderate pollution |
| Ⅴ |  |  | *P_Com_* > 3.0 | Strong pollution |

Li QH, Li CF, Wang H, Wei X, Liu, YS, Yang RD, et al. Geochemical characteristics of heavy metals in soil and blueberries of the core Majiang blueberry production area. Bull Environ Contam Toxicol. 2021;106:57–64.

**Table S8.** Correlation analysis between soil heavy metals and physicochemical properties.

|  | **pH** | **SWC** | **SOC** | **TN** | **TP** | **NH_4_^+^–N** | **NO_3_^-^–N** | **TK** |
| --- | --- | --- | --- | --- | --- | --- | --- | --- |
| Zn | 0.04 | -0.04 | -0.09 | -0.03 | 0.18* | -0.01 | -0.03 | -0.11 |
| Cu | 0.02 | -0.03 | -0.01 | 0.07 | 0.07 | -0.31** | -0.01 | -0.29** |
| Mn | 0.05 | 0.30** | -0.28** | 0.02 | -0.32** | 0.28** | 0.04 | 0.20* |
| Pb | -0.08 | 0.01 | -0.03 | -0.13 | 0.02 | 0.02 | -0.08 | 0.10 |
| Cr | -0.14 | -0.12 | 0.06 | -0.09 | 0.35** | -0.11 | 0.09 | -0.18* |
| Cd | 0.11 | -0.06 | 0.09 | 0.15 | 0.03 | -0.03 | 0.06 | -0.18* |
